# The New York Institute of Stomatology

**Published:** 1898-05

**Authors:** 

**Affiliations:** The New York Institute of Stomatology


### Reports of Society Meetings.

### THE NEW YORK INSTITUTE OF STOMATOLOGY.

A REGULAR meeting of the Institute was held Tuesday evening, March 1, 1898, at the residence of Dr. W. E. Hoag, No. 8 East Forty-third Street, New York City, the President, Dr. E. A. Bogue, in the chair.

The minutes of the previous meeting were read and approved.

Dr. J. A. Bishop exhibited an under plate which he thought had been worn about thirty years; he showed also a wisdom tooth that he had taken out with a string a few days before. This tooth had a large gold filling which was put in about thirty years ago by one of Dr. Atkinson's fine operators. The dentist had supported his gold filling with a little gold screw anchored in one of the roots, and the end of the screw extended beyond the apex of the root.

*The President.*—I believe some committees are to report. Has Dr. Allan, of the Committee on Operative Dentistry, anything to report?

*Dr. George S. Allan.*—Cataphoresis, which came into favor two or three years ago with a great flourish, has occupied much attention and consumed a great deal of time. While it has not gone entirely out of favor, it has seemed to fall behind, and many operators who at one time were enthusiastic about it, if asked if they use it as much as they formerly did, will frankly say, "No." The main reason given by most of these operators for giving up in some degree its use has been that it takes too much time and gives too much trouble. Most of the bother and trouble comes from the lack of quickly applied electrodes; therefore any electrode which meets the want indicated, which facilitates the use of the electric current in cataphoresis, ought to be known at once and exhibited. So far as I know there has been no advance made in the electrodes for the negative pole of the battery since their first introduction until this one was brought to my notice a few days since by Dr. Waite, of Waite & Bartlett. It is the result of the combined ingenuities of Drs. Kells and Van Woert, of Brooklyn, and is simplicity itself. It consists of U-shaped tongs, one end of which is a platinum bulb and the other a simple piece of rubber. The arms are about three inches long, the platinum bulb being about one inch long, one-fourth inch wide, and one-eighth inch thick. The mode of application is simply to put the platinum bulb against the inside of the cheek and the rubber on the outside. It does away at once with all bother of placing the cathode on the wrist or side of the face or elsewhere. It struck me as so valuable that I decided to bring it to the notice of the members of the Institute.

Another point Waite & Bartlett have introduced—whether they were the first or not to do so I cannot be certain—is having the two wires attached to the negative and the positive poles combined for most of their length, so as to do away with the annoyance of two separate wires. The double cord is composed of two cords, the green and the red, and they separate where they connect with the battery, and they separate again at the other end. A suggested improvement is that this cord be attached to a wheel that will unwind and wind on the principle of an ordinary curtain shade. The battery being in front of the operator, when he wishes to use it he simply pulls the rubber cord forward as he would pull down a shade, springing it back when he is done with it. In addition, I wish to show an electrode which I devised some time ago and have found exceedingly useful for attaching the anode pole to the teeth, so that both hands may be free. The anode end of the cord consists of a platinum wire which passes through a socket and can be clamped in position by a set screw. The socket in turn is attached to a bar with a flat pad below it. In use the pad is covered with modelling composition, and this last, after softening, is tied by a ligature to the adjoining teeth. All is then fixed in place. These little things make the use of the cataphoric current much more certain and greatly shorten the time of getting ready.

*The President.*—Will Dr. Allan kindly inform us how soon insensibility takes place after those electrodes are in position?

*Dr. Allan.*—In from ten minutes to half an hour.

*The President.*—Will Dr. Allan also inform us whether insensibility is induced cataphorically when the cavity is exceedingly shallow, barely penetrating the enamel?

*Dr. Allan.*—Yes, but very slowly. One cannot always tell by the symptoms the patient manifests whether the current is doing its work or not. Oftentimes the physical symptoms are wholly negative.

*The President.*—The Committee on Current Literature will now report through its chairman, Dr. Elliott.

REPORT OF COMMITTEE ON CURRENT LITERATURE.

*Dr. W. St. George Elliott.*—Among the most important questions which have arisen in the last six months are those introduced in the pathology of enamel by Drs. Andrews and Williams. It is desirable that we should have a clear comprehension of the points at issue between these two gentlemen. It has been noticed that our distinguished Society received a rather severe rap in the last number of the *Dental Cosmos* from our friend Williams, who thinks we assume to be the profession.

It would seem that the fundamental difference of opinion between these gentlemen is largely one of nomenclature. Dr. Andrews defines the enamel organ proper, when the organ is in its perfect state before calcification commences, and that a plexus of blood-vessels is not developed in an epithelial mass, while Dr. Williams speaks of the developing blood-vessels appearing in the stratum intermedium at an early stage in the formation of the tooth, or the enamel organ proper of Dr. Williams is the organ after calcification commences.

They both agree that the blood-vessels are developed from the connective tissue of the jaw, and that they are found in and near the stratum intermedium after calcification commences. Dr. Williams considers the stratum intermedium to be a glandular structure, while Dr. Andrews does not. Doubtless they differ as to what a glandular structure is, for they both agree that the apparent function of the stratum intermedium is to select nutriment from the blood for the use of the ameloblasts. Dr. Andrews holds the opinion in regard to the outer ameloblastic membrane, that it is not always present and may be a post-mortem appearance, while Dr. Williams generally finds this membrane in his specimens, and thinks it may be due to activity of the cells of the stratum intermedium beyond the capacity of the ameloblasts to absorb the material for calcification. They both agree as to the physical character of the structure; that it is a collection of fibres containing another substance in their meshes, which by one is supposed to be the protoplasm of the cells, by the other to be a product of the protoplasm. As to the inner ameloblastic membrane, one calls it the epithelial protoplasmic fibre, and the other plasm string.

They agree that the membranes, when present, are of a fibrous nature, having the appearance of a distinct substance and showing a definite reaction to certain stains.

Globules, which go to make up the enamel rods, are found in the ameloblasts. One gentleman calls them calcific globules, and the other enamel globules. They are deposited in, or there is simultaneously another substance formed, called by one protoplasmic exudate, by the other calcoglobulin, which forms in one instance the inter-rod cement, in the other the inter-prismatic substance. The organic scaffold is formed in one case by the epithelial fibre, in the other, plasm string. The steps in the process seem very much the same in both cases.

In the *Dental Digest* for January Dr. G. V. Black has a paper on Instrument Nomenclature, in which he has made a bold attempt to classify all our instruments. Whether his views will ever be generally adopted or not is a question. Yet the mere agitation of the matter will do much good. Let us hope that even we may live to see the day when students will be taught not only what instruments to use in a given case, but just how they should be used. Skill is a matter of labor and study. Mechanics are not usually made in a day. Some are born, but the vast majority acquire skill only by much patient effort and close study. The fault with most dental colleges in this matter is that more attention is paid to the shaping of the cavity than to the proper instruments to be used in its preparation and the proper manual with the instruments. The great majority of the profession develop a system of their own, and, good or bad, they stick to it through life, for, perchance, they know no other. The time taken to perform a specified operation varies with the operator, and is often two or three hundred per cent. Why is it? Two men will perform the same identical operation. One will take twice as long as the other, and why? Because there is no uniformity, no system in our procedure, no proper training. One uses a hoe, another a hatchet, and a third an engine, to do the same thing; and yet there is but one best way. Pardon this digression. Dr. Black would classify instruments and divide them into order names, as excavators; suborder names, as hand-plugger; class names, as hatchets; subclass names, as bayoneted plugger. Right and left hatchets he calls double-plane instruments, etc., and when the good doctor tells us of triple-angle-contra-angle, we get lost.

Some fifteen years ago I read a paper before a society at Ostend, Belgium, on "System as applied to Instruments and Books," in which I described the system I then used and now use. Briefly, with instruments it consisted of confining myself chiefly to hoes and hatchets, right angle and oblique, ten sizes of each, making a total of forty. All right angles were ninety degrees, and all obliques were forty-five degrees. These are in racks, and as the handles are in colored celluloid, the character of the instrument is seen at a glance,—red for hoes, and blue for hatchets. Pluggers are too complex in character to reduce them to a practical system.

In the *Dental Cosmos* for August, in the proceedings of the Dental Society of the State of New York, there is a report of the committee on Dr. Black's work, which is most interesting. Twenty-four fillings of amalgam were made by different operators; these were tested under Dr. Black's supervision, with the following results: three were perfect, sixteen shrank, and five expanded. Shrinkage was from one to sixteen points, Dr. Black's point being the $\frac{10}{1000}$ of an inch. The microscope (one-half inch objective) was used as a check. Should an error arise in the manipulation, it would probably be detected by this means. Two of the operators used gold to take up the excess of mercury and prevent shrinkage, which it failed to do. Dr. Black has accomplished two things, but think of the amount of work and the years of time it has taken to accomplish this! He has experimentally proved what are the proper metals and what the proper proportions of each, and that all alloys should be annealed. The proportion of silver and tin is seventy-two to seventy-four per cent. of silver and the balance tin, and that the annealing should be at 120° for three or four days, this giving a more plastic mass than when the annealing is done in boiling water for fifteen minutes. One of Dr. Black's own formulæ, silver, 68½; tin, 25½; gold, 4; zinc, 1; and bismuth, 1, gave perfect results.

An important question arises here as to how much contraction may be considered unimportant. Dr. Black's instruments measured up to the $\frac{1}{10,000}$ of an inch. But any one who is at all accustomed to work with even the B. & S. micrometer, measuring with fair accuracy up to $\frac{1}{4000}$, will recognize the skill required to work up to $\frac{1}{10,000}$. The average micro-organism, we will say, is about the $\frac{50}{1000}$ of an inch, while the instruments would fail to detect a shrinkage of less than $\frac{1}{10,000}$. Surely the organisms could hardly ask for more room, a more open door than they might have, and yet the margins pass muster as being perfect.

While this is true on theoretical grounds, do not forget the great gain we as a profession have made in having the shrinkage of our amalgam reduced from eight and ten points to nothing.

As to the proper amount of mercury to use, Dr. Black's rule seems a good one, and experimentally it has proved to be so. Use mercury enough so that the mass will take a good impression of the skin markings.

Why is it that we as a profession are so often treated by the manufacturers of amalgam as if we were children? Each labels his production as perfect, neither shrinking nor expanding, etc., whereas they know or ought to know that they all are unreliable, all shrink. Some years ago I tested by the specific gravity test some fifty different makes, and they all shrank. If they will use a shrinking formula, then let them give us the shrinkage ratio, and we will know what we are using. We do not want any *proprietary* make. We want the proper formula, and then give us the guarantee of a reliable house. Few dentists care to make their own alloy, and they are willing to pay a good price for a good thing. We are tired of *empirical* preparations. I might say, finally, that Dr. Black tells us that the amount of mercury present in a filling plays no part in the shrinkage, but a considerable one in the resulting strength.

In the same number of the *Dental Cosmos* Dr. Gillet has a paper on the subject with which his name is so frequently connected,—Cataphoresis. He drew attention to the modified views now held by the profession. Not only do not all medicaments go from the positive to the negative pole, but some go one way and some another. We can readily test the matter for ourselves by using a bit of moistened paper or cloth and placing the material to be tested at each pole and note the direction the color travels, or, if there is no color, by chemical tests. There is practically no improvement in the time required to reduce sensitiveness, from five minutes to half an hour. Secondly, dentine seems to act as a non-conductor. While on this subject I would like to mention the excellent paper by Dr. Price, of Cleveland. The first part of the paper seems to be largely given up to a scientific refutation of the so-called short circuiting the nerve. A matter of importance has been brought out by the doctor's experiments, that with a given current and a given pressure the result was the same whatever the electrode, the pain limit being reached independently of the character of the electrode. Also that different combinations of electrolytes gave the same results.

In the *Dental Cosmos* for November Dr. Stowell has an article on Hydronaphtol. Clinical experience certainly commends this ideal antiseptic. While fourteen times as strong as carbolic acid, it is neither irritating nor poisonous. It is of great value to us in many ways; at the same time, we cannot take the rosy view of the matter that Dr. Stowell does, nor do we believe that any drug or any system of treatment can be always successful. Oliver Wendell Holmes used to say, "There is no specific in medicine, if we except quinine in intermittent fever," and it is certainly true in dental medicine.

In the *Journal of the British Dental Association* of December 15 the Dental Manufacturing Company illustrate a slide-backing like the Mason, but sold separate from the tooth, and is thus made universal.

There has been in use in England for some time an apparatus invented by Dr. Hewitt, and now largely used abroad; it is for the combined administration of nitrous oxide gas and oxygen; its value has been most thoroughly proved; but as far as I know it is not used in this country. Again, why is it that in England for many operations outside of dentistry gas is first administered and then ether, getting largely rid of the stimulating and nauseating character of the latter when used alone? We are certainly a conservative people.

*Dr. S. E. Davenport.*—It has been noticed that the President, in sending out his list of the Institute's special working committees for the year, has added the names of a number of associate members. Several of the active members, in talking this matter over, have declared it to be a very good idea, as it will cause the associate members to take more interest in the scientific work of the Institute. I will therefore offer the following:

*Resolved*, That the Corresponding Secretary be requested to send to all associate members the request that they inform the President which committee they prefer to be appointed to.

The resolution was carried.

*The President.*—I will say that a number of the active members have earnestly requested that such steps should be taken as should cause the associate members to feel that they are on the same level as the active members except in matters appertaining to the government. I am in hearty sympathy with this action, and pleased that the Institute endorses it. It is with pleasure that I announce that Dr. Lord will now describe his method of using soft foil, and give us such demonstration of the same as he well can out of the mouth.

(For Dr. Lord's paper, see page 265.)

*The President.*—When Dr. Lord uses the word contour, I suppose he does not mean that he restores by absolutely non-cohesive gold a corner, for example?

*Dr. Lord.*—No; I do not mean that I restore a corner with soft gold. I said in my paper that cohesive gold would be required for restoring a corner or the side of a tooth that had been broken away. In my judgment, fillings are contoured too much beyond what is natural, professedly to prevent the food getting between the teeth; but food will get there, and the secretions also; and as the spaces cannot be thoroughly cleansed bad results follow.

*Dr. Kimball.*—Are these instruments with grooved lines on them made to draw or to push?

*Dr. Lord.*—Both; they will cut equally well by drawing or pushing. I have the instruments with me that I have spoken of this evening; but it is not easy to describe some instruments on paper, or even to understand them when they are illustrated. They must be seen and handled to be appreciated or understood.

*Dr. McNaughton.*—Will Dr. Lord tell us whom he recommends as a good instrument-maker?

*Dr. Lord.*—One of the best I have ever known is a man named Meffert. He is now in the employ of the Consolidated Company, and it is more difficult to get his services than when he had a shop of his own. It is very difficult to get suitable instruments made these days, and there is no way but for dentists to be able to make quite a good many of their own, as they must always be shaping the points in order to have just what they want.

*The President.*—It seems to me that our *confrère*, Dr. Kimball, did not quite finish his demonstration at the February meeting, and perhaps he will favor us with some comments on Dr. Lord's communication and finish what he was about to say the other evening.

*Dr. C. O. Kimball.*—I can hardly say that I did not finish the other evening, because what I read in the little outline paper really covered the ground. I should, however, have liked a little more time for the demonstration, to explain various little matters of detail, just as Dr. Lord has done here, not merely doing them, but explaining why they were done, but owing to the lateness of the hour I had to crowd through as rapidly as possible. The few who were near could see, but those farther away had to take it on faith.

I have listened with the greatest interest to Dr. Lord's explanation and demonstration. It seems to me a wise, intelligent, and thoughtful system, which he has worked out with great care, and I wish to express to him my personal gratitude for so fully and carefully giving us the details of it. I think Dr. Lord is right; our work is essentially a work of detail, and the successful man is the man of infinite patience and infinite detail. I mean the successful man, not from the business point of view, but from the professional stand-point. And so, when any man comes before us with an illustration of his method of work, we must expect and we want the finer particulars, because it is in those that we differ. We have a cavity, we pack gold in it, but in the minute details of the work are to be found the difference between one man and another.

It is a little difficult to criticise, to discuss clearly Dr. Lord's method without having seen his instruments before and without knowing exactly how each one is used, but the general plan of using the soft foil, of pressing it into the tooth against the walls of the cavity and then condensing it from the surface, I thoroughly approve of, as shown in my own practice, working on the lines of Dr. Dunning's teaching, though of course modified by my own individual feelings and habits of manipulation. Dr. Dunning usually packed every piece firmly as he put it in, and while in some cases I have followed the method Dr. Lord uses, it has seemed to me better usually to condense (to pack, as I call it) the individual pieces of gold firmly and about as solidly and carefully as it can be done with the first direct pressure at the time of putting them in. It is true that it leaves a rather hard surface, but the hardness of the surface of soft foil packed in that way is not so great but that the next piece may be made to stick to it. We call it non-cohesive gold, but I could show many fillings which have been in the mouth for years, and which when taken out showed a surprising amount of cohesion, and where nothing but pure soft foil was used. Dr. Dunning used to say that one of his friends carried in his pocket a gold eagle with a little filling built up on the side of it, using nothing but Abbey's soft foil and fine, sharp points. I have not seen the piece so treated, but it illustrates what can be done with soft foil. We do get a certain amount of cohesion by the working of the gold into the pieces already placed in position.

As for surface condensation, I feel that I am quite at one with Dr. Lord, for when the gold has been packed towards the walls carefully in the first insertion, the condensation does a twofold work. In the first place, by the wedging process in condensation the gold is packed against the walls still more firmly; and in the next place it affords, what is of great value to me, a thorough testing of the filling. I may put in a filling just as carefully as I can, and yet on going over it I find several places where I have to add more gold. It is that going over the surface and condensing and testing every corner and edge of the filling by which I feel sure when I get through that the surface is solid from edge to edge and that I have achieved the result at which I aimed,—to furnish a water-tight covering for that cavity. It is a cork, nothing more, hard enough to bear mastication.

In condensing, there is one instrument that I use a great deal which Dr. Lord does not, and which I should be very sorry to be without. It is a little bur with a tiny ball head, cut sharp, with a round handle so that it does not chafe the hand. In the middle of this round handle is a section which has a roughened surface. It is tempered just as a plugger is, and I can put any pressure on it that I want to, condensing with the little head, spreading the laminæ to the side until the surface becomes more or less hard. These tiny burs work by rolling the end (forward and backward) over and over, working from the centre to the side and from the side to the centre, being careful not to chip the enamel. This instrument is useful also in the packing of crystal gold; it works rapidly and effectively. A few years ago a friend of mine was filling a tooth in connection with another operator; one was using a serrated point plugger, filling one side of the cavity, and the other was using this little bur, working in crystal gold upon the other side. Years afterwards my friend had occasion to cut through that same filling, and he told me that the side which was packed by the little bur was much more solid and more closely condensed than the other side, which had been packed with the serrated point.

The only other thought I wish to refer to now is the use of the burnisher in filling. In my hands it is a very valuable instrument. I do not, however, use it of the size commonly employed, but much smaller, the head being a ball smaller than the size of the No. 1 bur. After the filling has been condensed with the square point and rolled over with the bur, this burnisher will rub the laminæ together still more, making a hard, smooth surface more impervious to moisture.

I would like to show this flat burnisher with an oblique turn. I have not seen it in any list of instruments, and have found, to my surprise, that it is a new thing to some of my friends. I have used it for many years. It is of almost universal application, the peculiar angle making it possible to use it for many purposes.

*Dr. Lord.*—I think I may have been misunderstood in regard to the use of the burnisher. I, of course, only had in mind that glossy finish which is so generally given to the surface of fillings and which is of no real value.

But to burnish in order to give further density to the filling and hardness to the surface is of the greatest value; indeed, it is imperative.

*The President.*—There are certainly a number of members who are accustomed to soft foil filling who ought to have something to say on this subject.

*Dr. C. B. Parker.*—This is a very interesting subject to me, because it is the school in which I was brought up. I do not know that I can add anything to what has been said here to-night by Dr. Kimball and Dr. Lord. My present method is a combination of their methods, and if I were to say anything it would be to simply repeat what they have said.

*The President.*—Dr. Hawes was expected to be present this evening, and has sent a written communication which I will ask Dr. Allan to read.

Dr. Allan reads:

"20 West Forty-seventh Street,
"New York, March 1, 1898.

"Dear Dr. Allan,—I regret very much that an engagement for this evening which I had overlooked will prevent me from being present at the meeting of the Institute. As you requested me in case I was unable to be present to write my views regarding the use of non-cohesive foil, I will simply say that for fillings on the approximal surfaces, which is the crucial test for all filling materials, it ranks in my opinion far above cohesive foil. It has, however, the disadvantage when hand-pressure is used, as so well described by Dr. Kimball at your last meeting, of causing the conscientious operator most laborious nerve- and strength-wasting labor. That continued spasmodic, nervous, mallet-like action of the arm has shortened the professional life of many a dentist, and while here and there some more vigorous than the rest have withstood it, yet it will show its effects in time. Knowing this by a personal experience, I began several years ago to look for some means less exhausting which would give as good results, and in my automatic plugger I found it. I have points similar to those shown by Dr. Kimball, but made a trifle heavier at the angle to prevent breaking. With these in my automatic plugger I can condense the soft foil laterally in all directions except that towards the hand in which I hold the instrument; much of the final condensing can be done with the same instrument, and the whole filling finished with infinitely more ease to patient and operator. All this I know would be looked upon as rank heresy by Dr. Dunning, but I feel it a duty to myself to make my work as easy as possible, when the standard of the work is in no way lessened. If I am not mistaken the early filling of teeth with gold foil was done exclusively with non-cohesive foil, and where the cohesive quality was required, Watts's crystal gold was used. This was true of the operations performed by Dr. Eleazer Parmlee before and after Dr. Dunning was associated with him as his assistant, using the same instruments described by Dr. Kimball. My father also used the same method many years ago, and I have now instruments used by him before Dr. Dunning's time which are precisely the same as those used by Dr. Dunning later. Please bear in mind that I want to say nothing derogatory or reflecting in any way on Dr. Dunning. He was a wonderfully clever operator, and the work of his predecessors would not compare in finish and elaborateness with his, but unless the mere using of a method makes it one's own, I do not see the propriety of calling this method Dr. Dunning's.

"Yours very truly,

(Signed) "JOHN B. HAWES."

*Dr. Allan.*—In reference to the burs that Dr. Kimball spoke about, I fully concur in all he said, but I would warn any one who tries them that he cannot use them with success until the inside of his thumb and forefinger have become toughened; it takes but a very short rolling of that instrument to cause quite a numb and calloused feeling, but it does pack the gold most beautifully, and to the patient it is a great comfort. Where a patient will grumble at an automatic mallet he will almost go to sleep if this bur is being used in packing the gold, but one has to practise a long time before he becomes accustomed to it and can properly use it. Dr. Hawes was at our last meeting, and, happening to ask him one or two questions in regard to the Dunning method of using soft gold, I found he had one or two points that were worthy of attention. The doctor was unable to be present to-night, but has sent the letter extracts from which I have just read.

One point only I would draw attention to. If Hawes has perfected a method of using points to pack soft gold foil as we pack cohesive gold foil and with comfort to the operator, it is a great point in advance.

The President, Dr. Bogue, called the Vice-President, Dr. C. A. Woodward, to the chair.

*Dr. E. A. Bogue.*—This matter of soft foil filling is one to which I also was brought up, and as there are one or two points possibly where my work has varied a little, not in principle but in practice, from that of the gentlemen who preceded me, I will venture to say a few words.

Dr. Hawes is, I think, mistaken in writing as he does about non-cohesive foil and crystal gold. If I am not in error, pure gold is cohesive, providing it is clean. The cohesive properties of gold-foil were discovered by the act of a druggist who, wishing to send a book of gold through the post-office to a dentist, slipped the leaves out and enclosed them in an envelope; the result was that the leaves stuck together and the dentist had to cut the gold off in strips. This shows clearly that the cohesive properties of gold existed before dentists knew anything about it. After these cohesive properties became better understood, a school of practitioners arose that discarded the wedging process of packing gold, discarded the processes about which we have listened with so much pleasure and profit this evening, and began from the very bottom of the cavity with a couple of divergent holes drilled into the teeth, laboriously to build up cohesively from that to the end. It has always seemed to me that this was a mistake. I had a lady in my chair Saturday for whom I filled teeth thirty years ago; that is a good while. Nearly all my fillings were still there. The bottoms of those fillings were put in with non-cohesive foil, and where the cavities were surrounded by walls the fillings were mainly put in with cylinders, like cigars standing up in a tumbler. Wherever those cylinders had been packed, as Dr. Kimball showed us the other night, by wedging backward and sideways towards the wall of the cavity, the fillings put in thirty years ago are to-day bright and shiny. Now, all my fillings unfortunately have not been that way.

When I lived in Chicago the question came up, and we had it hotly discussed, how to put in fillings that would not pit upon the surface. The cohesiveness of gold was more or less understood at that time, but there came to us in Chicago Dr. Clark, of New Orleans, and Dr. Knapp, the father of Dr. J. Rollo Knapp, both of whom had learned cylinder filling from a gentleman in Alabama whose name I have now forgotten. Their processes were in their results identical with the processes of which we have heard this evening and at our last meeting,—namely, to cause the gold to lie in laminæ parallel with the walls of the cavity and to have the gold finished off at the end precisely as the leaves of a book might be planed off, leaving a smooth surface. Later on, after this Chicago lesson which I had taken to heart,—viz., the fact that all gold is cohesive if it is clean and pure,—recognizing that what we call non-cohesive gold is simply gold with something on the surface, I adopted the practice of filling the bulk of my cavities, where they could not be filled with cylinders, with what was known as non-cohesive gold, and when I had reached a certain point where I wanted a smooth surface, if I was unable to adopt the principle of parallel laminæ and cutting off the ends, then I used cohesive gold and drove it directly into this non-cohesive gold, with full confidence that it would stay there. Generally, by interdigitation and relying upon the cohesive qualities of the central portion of each leaf, we can be certain that we have a good anchorage. If we want a cohesive gold surface, we can safely add it to a non-cohesive filling.

*Dr. F. Milton Smith.*—I would like to ask Dr. Bogue in reference to the fillings whether he finished them with a cohesive foil. He said he filled the bulk of the cavities with non-cohesive foil.

*Dr. Bogue.*—The cylinders were so filled that they did not need the cohesive foil, but cavities needing contouring had cohesive foil on the surface.

*Dr. Lord.*—If cohesive gold is to be used for the surface of fillings there must certainly be a place for starting it; it must be secured mechanically, as it will not cohere to the soft gold. If the soft gold has not been packed hard and has been left so that the cohesive gold can be forced into it, that will do for an anchorage, but I much question the propriety or usefulness of cohesive gold for such a purpose.

*Dr. C. S. Stockton.*—We seem to be in the old barn to-night, threshing over the old straw, getting out some wheat perhaps, but it takes us back a great many years. Like my friend Dr. Bogue, I began with soft foil, but recognized, as many others do to-day, that the old Concord coach is a slow way to travel, and I prefer the trolley and cable. Things are moving in a more rapid way than they did a few years ago and we have to keep up with the times. To-day I saw a gentleman who had a tooth filled fifty-seven years ago, and the filling has remained there just as put in. It was filled, of course, with soft foil, and he has been so prejudiced in favor of gold that he will not have his own or his children's teeth filled with anything else. Some time ago one of his daughters had a wisdom-tooth to be filled, the mouth being very small, and I think that by the time she got through she was willing to have wisdom-teeth, at least, filled with other materials than gold.

Dr. Bogue has been kind enough to give us a bit of personal experience, and I hope I will be excused for following his example. Two months ago a Sister of Charity came in. I took my glass, looked over her teeth, and very innocently said, "Sister, you have had some good work done on your teeth." I saw a kind of smile pass over her face as she said, "Doctor, you don't recognize me?" I said, "No, I do not." She then said that in September, 1872, I had put all the fillings in her teeth. Those teeth were filled with soft foil, finished with cohesive gold. Twenty-five years is a pretty fair test, and those fillings are in as good condition now as the day they were put in. Is it not wonderful that with these metal patches we are able to preserve teeth as long as we do,—twenty-five, thirty, and even fifty-seven years? Yet some patients come in and complain because a filling has come out. We have done the best that man can do, and yet they expect us to do better for them than the Lord did for them in the beginning.

*Dr. Smith.*—I am free to say that I have not attained to that degree of proficiency that I should like in the use of non-cohesive foil. I am trying hard and am meeting with some success. I have filled many cavities with non-cohesive foil, but in some cases when I come to finishing, not having the centre of the filling thoroughly condensed, I have used for the surface the gold as it comes in the bottles, oftentimes much softer than when we pass it through the flame, and yet quite cohesive. I have worked that into the centre of the filling, and am sure I have made successful fillings in that way.

The report of the committee upon the death of Dr. C. W. Meloney was then presented, as follows:

WHEREAS, An all-wise Providence has seen fit to remove from among us our friend and fellow-worker, Dr. Charles W. Meloney; therefore,

*Resolved*, That with becoming reverence we most humbly submit to the will of Him who knows the end from the beginning.

*Resolved*, That we recognize in our departed friend and brother one of those rare characters with whom this world has not been too bountifully supplied.

*Resolved*, That in his upright and manly course as a member of our profession, never seeking preferment at the expense of others, but rather standing in the background and enjoying their elevation even at cost to himself, we recognize that true manhood which is exemplified in the words of Scripture, 'Is not puffed up, seeketh not her own, is not easily provoked, thinketh no evil, rejoiceth not in iniquity, but rejoiceth in the truth.'

*Resolved*, That in the death of Dr. Meloney we have lost an honored friend, the Institute a faithful member, and the world that which is more rare than it should be, a faithful, conscientious dentist.

*Resolved*, That as a Society we extend to his companion, Mrs. Meloney, and their children our most sincere sympathy, and commend them to the gracious care of that kind Providence who careth for all His children.

*Resolved*, That a copy of these resolutions be sent to the family of Dr. Meloney and also spread upon the minutes of the Institute.

(Signed) F. Milton Smith.
J. G. Palmer.
S. H. McNaughton.

The report of the committee was unanimously adopted.

Adjourned.

S. E. Davenport, D.D.S., M.D.S.,
*Editor The New York Institute of Stomatology.*

